# Friends close, enemies closer: the complex role of the microbiome in antitumor immunity

**DOI:** 10.1016/j.coi.2025.102537

**Published:** 2025-02-27

**Authors:** Dipyaman Patra, Gagan Dev, Timothy W Hand, Abigail Overacre-Delgoffe

**Affiliations:** 1 Department of Immunology, University of Pittsburgh School of Medicine, Pittsburgh, PA 15213, USA; 2 Tumor Microenvironment Center, UPMC Hillman Cancer Center, USA; 3 Department of Pediatrics, UPMC Children’s Hospital of Pittsburgh, University of Pittsburgh School of Medicine, Pittsburgh, PA 15224, USA

## Abstract

Immunotherapy has achieved remarkable advances in cancer treatment by harnessing the immune system to combat tumors, yet its effectiveness remains inconsistent across patients and tumor types. The microbiota, a diverse assemblage of microorganisms residing at host barrier surfaces, is pivotal in shaping immune responses. This review explores the direct and indirect mechanisms via which the microbiota modulates antitumor immune responses both locally within the tumor microenvironment and systemically by affecting distant tumors. We discuss recent findings linking microbiota-derived metabolites and microbiota-derived antigens with antitumor immunity and immunotherapy response. Additionally, we discuss recent advances in microbiome-based therapies, including fecal microbiota transplantation. We propose the use and development of new analytical techniques to further characterize the complex functions and interactions between the microbiome and immune system. To conclude, we outline recommendations for future research and therapeutic approaches to leverage the microbiome to improve current immunotherapies.

## Introduction

Huge breakthroughs in immunotherapy, where immune cells are unleashed to kill tumors, have revolutionized the treatment of many forms of cancer. Unfortunately, these therapies are far from universally effective, and there is a critical need to understand the host and environmental factors that drive treatment efficacy or failure in different patients and various tumor environments.

Among the environmental factors that are most important to the development and function of the immune system is the microbiota. This collection of bacteria, fungi, protists, and viruses that inhabits host barrier surfaces are not random assemblages but instead are specific communities that provide critical functions. The microbiota can affect the immune response in at least two different ways. The first is that the microorganisms of the microbiota share many of the microbe-associated molecular patterns with pathogens and thus can activate immune cells via stimulation of pattern recognition receptors (PRRs) [1••,^2^]. The second is that the microbiota produces a wide variety of metabolites that can affect immune cells broadly through multiple mechanisms [3]. The microbiota represents a tantalizing therapeutic target because drugs would likely have little overlap with host enzymes and modulation of the microbiota via ‘fecal microbiota transplant’ should be reversible. Thus, it is not surprising that augmenting the microbiota to activate antitumor immunity has been an active area of research. Connections between bacteria, viruses, and fungi have been made to a variety of antitumor immune functions [4,5]. Excitingly, there have already been successful translations to the clinic, where it has been demonstrated for melanoma that some treatment-refractory patients can be induced to respond to checkpoint blockade therapy after fecal microbiota transplantation (FMT) from patients who have responded to therapy or from healthy donors [6••,7••].

Despite these positive developments, there is still much to learn about how we may be able to harness the microbiota to improve immunotherapy. For example, while multiple studies have now identified microorganisms associated with response to anti-programmed cell death protein 1 based melanoma therapy, the bacteria identified did not overlap, and even using metagenomics, a shared set of microbial genes or functions that must be present or enriched intestinal microbiota for treatment success was not obvious [8]. In some instances, the same bacterial taxa tested in separate laboratories have been described to either support or diminish immunotherapy. We would argue that as we move forward, it is paramount for the field to adopt modern approaches that will help to reconcile these disparate findings and identify microorganisms and configurations of the microbiota that will support immunotherapy success in a broader swath of patients. Indeed, more recent approaches have revealed potentially predictive microbiota ‘signatures’ for increased survival during anti-PD1 immunotherapy [9••], but whether this signature can be generally extended to global patient populations or other tumor immunotherapies has yet to be determined. Our recommendations for improving the universality of microbiota findings are included in Box 1.

The intestine is home to the largest and most diverse microbiota and intestinal bacteria have been most closely studied for their effect on antitumor immunity, though fungi and viruses can also be very important [10,11]. Colon cancer is common, and much research has focused upon how the microbiota modulates tumor growth and immunity locally. However, metabolites and other products of the microbiota can be taken up by the host and transported to distant sites to affect antitumor immunity systemically. The microbiota can also activate T and B cells directly through antigenic stimulation. Finally, certain types of bacteria typically found at barrier sites can travel to and grow within tumors themselves, forming a tumor microbiome. Here, we will discuss how the microbiota affects antitumor immunity to both local and distal tumors, both through metabolites and direct antigen-mediated activation of T and B cells. A major goal for the field will be to synthesize how different microbiota-derived signals modulate antitumor immunity in varied tumor and host environments. Given the complexity involved, it is likely that machine learning approaches will be necessary to harmonize the data into a comprehensive model. In the absence of such a synthesis, here we will present individual examples to demonstrate the mechanisms by which the microbiota can affect the antitumor immune response. In the last section, we will focus on one genus (*Lactobacillus*) that is commonly linked to antitumor immunity but with highly diverse outcomes depending upon the context of the host and tumor environment.

### Microbiota-derived metabolites and cancer immunity

Metabolites from the gut microbiota play a crucial role in cancer modulation by influencing the tumor micro-environment (TME) and signaling pathways in both cancer cells and immune cells. Major metabolites produced by the microbiota include short-chain fatty acids (SCFAs), secondary bile acids, polyamines, and tryptophan-derived metabolites. Microbiota-derived metabolites have been associated with both tumor progression and suppression functions. Paradoxically, some microbiota-derived metabolites can both support or inhibit tumor growth likely because metabolite receptors are expressed upon different cell types and have pleiotropic effects.

### Effects of the intestinal microbiota-derived metabolites on immunity to colorectal tumors

**Figure 1:**
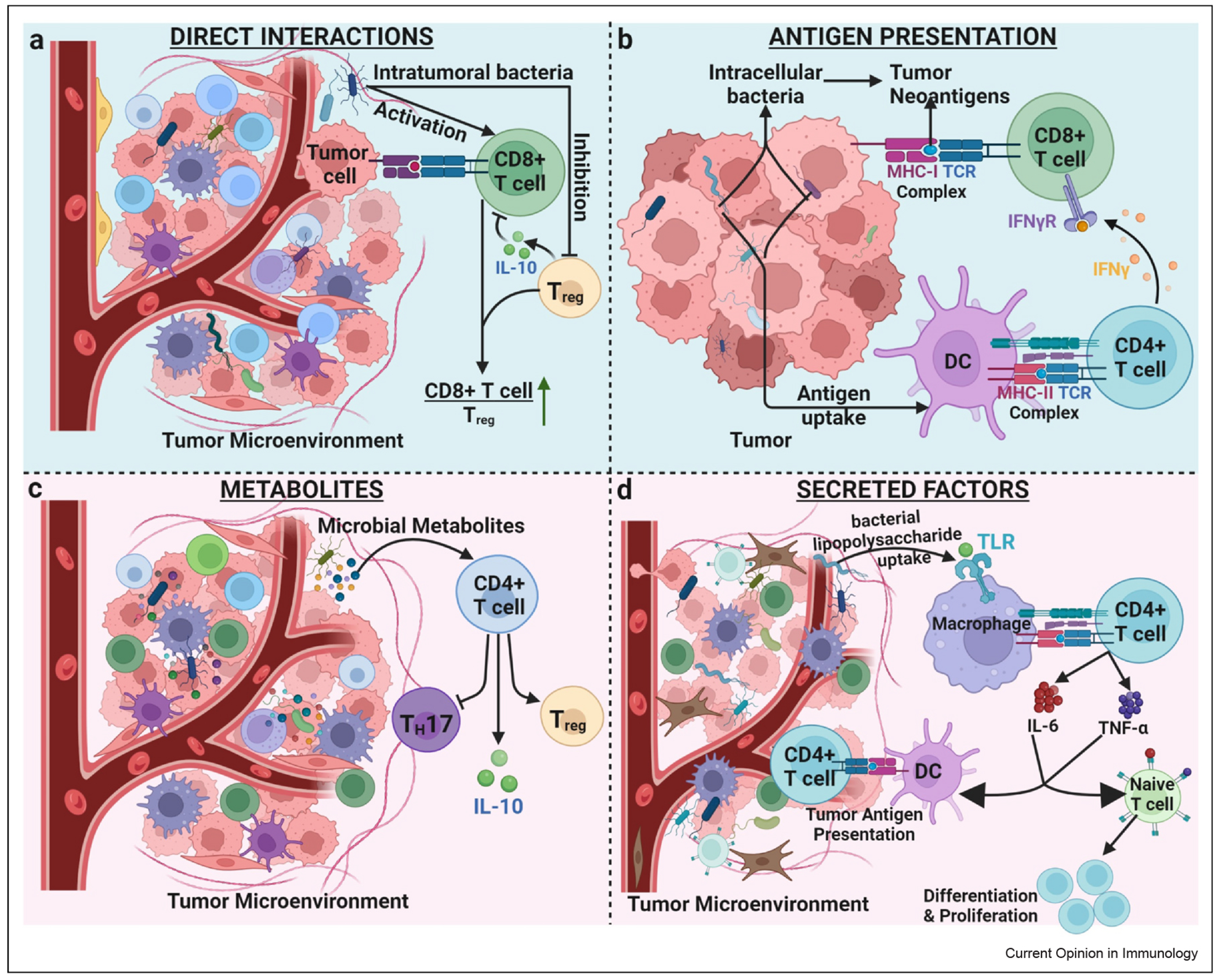
Mechanisms of immune modulation by commensal tumor microbes in the TME. **(a)** Direct interaction of microbes with resident T cells leads to an increase in CD8+ T cells/T_regs_ ratio intratumorally. **(b)** Antigens from intratumoral microbes can get processed by either MHC-I or MHC-II on the surface of tumor cells or DCs to facilitate activation of CD8+ T cells and enhance tumor clearance. **(c)** Microbial metabolites generated in the TME of CRC patients interact with CD4+ T cells to influence their differentiation and protect against colitis-induced cancer. **(d)** Bacteria lipopolysaccharides get taken up by macrophages in the TME and lead to release of cytokines such as IL-6 and TNF-α, which leads to immune differentiation and proliferation in naïve T cells. MHC, major histocompatibility complex; TNF, tumor necrosis factor.

Given the proximity and huge enzymatic functionality of the intestinal microbiota, it is perhaps unsurprising that it has a critical function in the development of colorectal cancer (CRC). For example, *pks^+^ E. coli* can generate mutagenic compounds that directly contribute to the development of CRC through the induction of DNA damage in the host epithelium [12]. The microbiota can also support tumor growth by modulating the immune system to induce inflammatory cytokines that drive oncogenic transcription factors, such as STAT3 [13–15]. Indeed, toxin-producing Enterotoxigenic *B. fragilis* (but not toxin-negative control bacteria) induces T helper 17 (Th17) cells that support colorectal tumor growth [16]. Other metabolites also shape the development of CRC through modulating tumor-resident immune cells. Colonic bacteria produce large amounts of SCFAs, such as butyrate from the digestion of dietary fiber. Butyrate is associated with reduced tumor burden in colon cancer through increasing differentiation of interleukin (IL)-10 producing T (Tr1) cells (Figure 1c) that suppress inflammation in the colon [17]. *Fusobacteria* colonization of colonic tumors is associated with reduced migration of lymphocytes to the tumor, increased migration of myeloid cells, and worse patient outcomes, all driven by local SCFA production [18,19,20•]. Secondary bile acids are major immune modulators produced by the microbiota through the modification of host-produced primary bile acids. Similar to SCFAs, secondary bile acids support the induction of colonic Rorγt-expressing Foxp3+ regulatory T cells (Tregs), which can suppress antitumor T cell response. In a recent study by *Cong et al.,* deoxycholic acid has also been shown to suppress CD8^+^ T cell responses through plasma membrane Ca^2+^/AT-Pase that inhibits nuclear factor of activated T cells [21•]. Using CRC as an example, we can see that multiple pathways involving microbiota-derived metabolites influence tumor growth and immune responses.

### Effects of intestinal microbiota-derived metabolites on antitumor immunity outside the gastrointestinal tract

The role of microbial metabolites in regulating antitumor immunity is not restricted to the intestine as metabolites can be absorbed into the bloodstream and traffic systemically. For example, immunotherapy can impair intestinal barrier function, leading to the translocation of bacterial products from the gut into the bloodstream. These products can influence myeloid cells in distant tumors, potentially enhancing the effectiveness of chemotherapy and immunotherapy by modulating the immune response in the TME [22]. Similarly, inosine, produced predominantly by *Bifidobacterium pseudolongum*, increased adenosine 2A receptor–mediated activation of tumor-specific T cells and enhanced antimelanoma immunity [23]. Additionally, the microbial metabolite tryptophan has been associated with enhanced immunotherapeutic response to checkpoint inhibitors [24]. Thus, for distal tumors, much like colorectal tumors, there is a plethora of mechanisms by which the microbiota can affect antitumor immunity, and the mission for the field is to find a way to integrate them all to understand what would most benefit the patient.

### Role of microbiota-derived antigens in modulating antitumor immunity

Beyond the substantial effects of microbiota-derived metabolites, each human intestinal microbiota can harbor up to 1000 different bacteria, fungi, and protists, each of which can have 1000s of potential antigenic proteins. Given the substantial diversity in microbiota-derived antigens, it is perhaps unsurprising that cross-reactivity has been discovered between the microbiota and tumors that can affect antitumor immunity. Zitvogel et al. identified that an antigen derived from bacteriophages present in *Enterococcus hirae*, which mimics a host-derived peptide, is crucial for activating antitumor, interferon (IFN)-γ-producing CD8+ T cells following cyclophosphamide treatment [11,25]. This is an exciting discovery as it leads to the obvious conclusion that tumor antigens could be mimicked to the host by organisms in the microbiota. This strategy has recently been demonstrated using engineered *Staphylococcus epidermidis* expressing melanoma antigens in the skin driving antitumor T cell response with compelling effects [26•].

Microbiota-specific T cells can also be important modulators of the immune response without directly activating antitumor immunity. In mice with colon tumors, *Helicobacter hepaticus* colonization leads to the induction of peritumoral tertiary lymphoid structures (TLS) that are dependent upon the generation of *H. hepaticus*–specific CD4^+^ T follicular helper T cells (Tfh). In this model, microbiota-specific Tfh were both necessary and sufficient for the generation of TLS and increased immune cell infiltration into tumors, leading to reduced tumor burden in mice. Microbial epitopes can also lead to the activation of tumor-resident T cells and CD103^+^ dendritic cells (DCs) to increase IFN-γ production and cytotoxicity of CD8^+^ T cells [22,27]. Understanding how antigens derived from the microbiota could be harnessed to activate antitumor immunity or, more provocatively, whether the organisms from the microbiota could be used as ‘vaccines’ against tumor growth is another exciting area to pursue in the near future.

### Activation of immunity in the tumor microenvironment by tumor microbiome

Under specific circumstances, bacteria from barrier tissue sites can populate tumors and other sites of disease and modulate local immune responses. How and why bacteria translocate from the barrier tissues to the tumor is still largely unclear. For some bacteria, the necrotic, acidic, and anaerobic core of the tumor provides a fertile environment for their growth [28,29], but other organisms may arrive in the tumor less actively. It is also not clear whether tumors foster the growth of specific bacteria to promote tumor cell growth and limit immunity or whether the host sends microorganisms to the tumor to produce the exact opposite. However, regardless of the governing mechanism, it is clear that selective modulation of local microbiome by introducing or eliminating certain tumor-associated microbes while preserving the balance of host’s natural microflora can improve antitumor immunity and increase the chances of long-term survival in patients with cancer [30,31•].

The types of microorganisms colonizing tumors are distinct between cancer types [32], such as pancreatic cancer [30], oral and colon cancer [33••], prostate cancer [34], breast cancer [35], lung cancer [36], thyroid cancer [37], gastric cancer [38], nasopharyngeal carcinoma [39], and renal cell carcinoma [40]. Tumor-associated microbes are partly similar to the adjacent normal tissue microbiome [41], but there is also evidence for the gut microbiota to colonize distal tumors. Additionally, environmental factors, such as diet, lifestyle, antibiotic use, chemical exposure, and geographical and socioeconomic factors, can alter the tumor microbiome and play an important role in shaping the tumor immunity [42]. Intratumoral microbes can impact the antitumor immune response directly through residing within tumor or immune cells and indirectly through secreted molecules within the local TME. Many intratumoral microbes are located intracellularly in both tumor cells and immune cells [32], which can directly stimulate the activation of immune cells, such as T cells, B cells, macrophages, and DCs, to drive or inhibit antitumor immunity [33••,^43^]. Interestingly, bacterial epitopes can be presented on the surface of tumor cells, further driving the antitumor immune response. This leads to HLA-I- and HLA-II-mediated activation of both CD4+ and CD8+ T cells in the TME, which can be crucial in aiding the CD8+ T cells in clearing the tumor and improving the efficacy of therapeutic responses (Figure 1b) [44]. Microbes can also have direct interactions with immune populations that ultimately can control the antitumor immune response. Examples of microbes altering the antitumor immune response include increasing in the ratio of intratumoral CD8+ T cell/T_regs_ [45] (Figure 1a) and activating local DCs within the TME, leading to increased antigen presentation of tumor antigens (Figure 1b). Microbes can also influence the chemokines and cytokines levels in the TME. For example, the lipopolysaccharides in bacterial cell walls can upregulate IL-6, IL-8, and tumor necrosis factor (TNF)α levels [46], thereby promoting immune cell proliferation and tumor antigen presentation via DC and macrophage maturation (Figure 1d). Other bacterial products like the peptidoglycan layer and bacterial polysaccharides can activate immune cells and lead to expression of cytokines such as IFN-γ, tumor necrosis factor (TNF)-α, IL-6, and IL-12 [47] that would promote antitumor immunity in the TME. Furthermore, intratumoral bacteria like *Lachno- clostridium* can stimulate the release of chemokines like CXCL9, CXCL10, and CCL5 and result in increase of CD8+ T cell infiltration in cutaneous melanoma [48].

Microbes can have deleterious effects on immune cells within the TME, most commonly through bolstering immunosuppressive cell populations, including T_reg_ cells and tumor-associated macrophages. Microbial induction of PRRs can activate immunity but conversely lead to the production of inflammatory cytokines [49] or the induction of anti-inflammatory regulatory immune cells [50,51]. For instance, intratumoral microbes can be recognized by Toll-like receptors TLR2 and TLR4, resulting in M2 macrophage polarization and recruitment of myeloid-derived suppressor cells, supporting an immunosuppressive TME [52,53]. Additionally, tumor-associated microbes can further drive an immunosuppressive TME through T_reg_ recruitment and CD8^+^ T cell exhaustion [38].

Considering the unique microbial and physiological composition of TME, the limited examples presented here only highlight the potentially immense role of microbes residing in the TME that remains to be uncovered. The prevalence, diversity, and functional characteristics of the tumor microbiome between cancer subtypes and even between patients remain unclear. Given the great variability of tumor-associated bacteria between patients, efforts to modulate this population for therapeutic gain may need to be highly targeted to and personalized to the patient and tumor.

### Case study: *Lactobacillus* and antitumor immunity

One bacterial genus, *Lactobacillus*, has been commonly associated with antitumor immunity across many studies, so deeper look at its varied effects could be illustrative of the level of complexity present in how the microbiota affects antitumor immunity. *Lactobacillus* is commonly found in the gut and at other mucosal surfaces and has been found to traffic to tumors in some models, so it represents an excellent model for the concepts discussed above. Illustratively, the impact of *Lactobacillus* on antitumor immunity can also be either protective or tumor promoting, depending on conditions such as tumor type, state of host immune system, and, perhaps most importantly, the specific strain and species of *Lactobacilli*. For example, *L. rhamnosus* GG can enhance CD8^+^ T cell–mediated immunity via activation of DCs in CRC [54] and *L. plantarum* can control CRC growth by enhancing tumor-infiltrating CD8^+^ T cells through the production of indole-3-lactic acid [55]. Additionally, production of indole-3-propionic acid by *L. johnsonii* promotes activation of exhausted CD8^+^ T (Tpex) cells in tumors during immune checkpoint blockade (ICB) therapies, thus improving the responsiveness of ICB at pan-cancer level [56]. On the contrary, overabundance of *Lactobacillus* species is associated with tumorigenesis in gastric cancer [57]. Moreover, while dietary metabolites derived from tryptophan by *Lactobacillus reuteri* can immune checkpoint inhibitor efficacy via AhR signaling in CD8+ T cells in melanoma [24], AhR activation of macrophages by the same bacteria can lead to immune suppression and tumor-promoting effects in pancreatic cancer. Deletion of the AhR receptor in myeloid cells promoted ICB therapy and increased the frequency of IFNγ-producing CD8^+^ T cells in the tumor [4]. Moreover, in the melanoma studies, many types of bacteria could provide the tryptophan-derived AhR ligands, but *L. johnsonii* could not, which is dissonant with the literature discussed above. Therefore, while some strong generalities can be drawn for specific bacteria and tumor types, such as the negative effect of *Fusobacterium* on colorectal tumors, the incredibly variable results associated with different species of *Lactobacillus* indicate that we have so much more work to do to understand how various bacteria modulate immunity in different tumors. In contrast, we can take some hope from the fact that many of the *Lactobacillus*-dependent mechanisms depend upon tryptophan metabolites (indoles) signaling through AhR, so approaches based upon metabolites/bacterial products may lead us to simpler answers.

### Methods for modulating the microbiota to improve tumor immunotherapy

Given the associations between the gut microbiome and immunotherapy responses in patients, there has been excitement for the concept of therapeutic modulation of the gut microbiome. The most prominent method, FMT, is where the gut microbiome from patients responding to immunotherapy or healthy donors is transferred to nonresponders in an attempt to reinvigorate their immune system to respond to immunotherapy [58–60]. In some circumstances, FMTs have been associated with increased density of intratumoral T cells and Granzyme B+ cells within the TME and long-term patient survival in PDAC [30]. FMTs also have been shown to introduce microbes that induce type I interferon production by intratumoral monocytes and improve the efficacy of ICB [61].

While promising, FMTs often fail, likely as a result of the fact that the exact consortia of bacteria that provide a benefit remains unclear across patients. Identification of potent, well-tolerated, antitumor organisms may vastly increase the efficacy of targeted FMT therapy. Ongoing targeted approaches include the identification of microbial peptides that cross-react with tumor antigens [11,62–64], design and delivery of tumor-mimicking microbial epitopes [65] via tumor-derived extracellular vesicles [66], or DCs [67]. Additionally, engineering bacteria to express tumor antigens have proven successful in preclinical models [28,68] or ligands for chimeric antigen receptors [69•] designed to kill the tumor. Considering the role of microbial metabolites in both promoting and inhibiting antitumor immunity, an indirect approach could involve selective administration or removal of certain microbial metabolites [70].

While the prospects of using commensal human microbes to bolster antitumor immunity are very promising, many challenges remain. As discussed, one of the major obstacles is the variation in the local microbiome both inter- and intra-individually, making it difficult to develop generalized prebiotic or postbiotic supplements that could promote antitumor immunity at a community level. In addition, external environmental factors (such as diet, lifestyle, age, gender, use of antibiotics, etc.) add even more variation to the limited knowledge of the diversity of human microbiome. Techniques to rapidly dissect the microbiome at an individual level remain limited to sequencing technologies that include amplification steps, further convoluting our understanding of the tumor microbiome (Box 1). We posit that a better understanding of the microbe:host immune system relationship as well as development of improved technologies to assess the functional microbiome at both a single organism and host level will aid in future therapies involving the microbiota.

## Data Availability

No data were used for the research described in the article.

## Box 1

### Microbiota 2.0.

#### Sequencing methodologies:

16S rRNA amplicon sequencing can be a useful tool to describe the overall structure of the microbiota from samples where bacteria are abundant, but this method should not be used as the sole method for identifying taxa. This method is not useful for identifying bacteria beyond the genus level and, because of the polymerase chain reaction amplification step, is particularly troublesome to apply to low biomass samples because the effects of contaminating bacteria, host cells, and concatemerization are magnified. When possible, shotgun metagenomic or metatranscriptomic analysis should be performed. Current sequencing technologies are challenging to perform quantitatively and in the future single cell technologies will likely be necessary to capture absolute rather than relative abundances.

#### Culturomics:

Most organisms of the microbiota can be cultured as parts of consortia, and attempts should always be made to isolate and culture the microorganisms identified by sequencing to be associated to a given host outcome. Live culture of the bacteria has many benefits, not the least of which is that it allows for easier measurement of bacteria-produced metabolites and, in some cases, genetic manipulation.

#### Imaging:

Multiple imaging modalities now exist for the identification of bacterial DNA (fluorescent in situ hybridization) and RNA (hybridization chain reaction) that can be performed on either sections or ‘optically cleared’ tissue samples. By use of co-staining of bacteria with multiple specific probes to each taxa even small populations of bacteria can be identified with confidence. New modalities (MER-fluorescent in situ hybridization, expansion microscopy) will only increase the utility of imaging to define microorganisms’ association with the host.

#### Other kingdoms of life:

The vast majority of research on cancer, immunity, and the microbiota has focused on bacteria, but we now know that fungi, protists, and viruses might also play important roles. More comprehensive ‘trans-kingdom’ analyses should be investigated.
